# Color stability of hybrid ceramics exposed to beverages in different combinations

**DOI:** 10.1186/s12903-022-02206-1

**Published:** 2022-05-14

**Authors:** Kaan Yerliyurt, Işıl Sarıkaya

**Affiliations:** Department of Prosthodontics, Tokat Gaziosmanpaşa University Faculty of Dentistry, 60100 Tokat, Turkey

**Keywords:** Beverages, CIEDE 2000, Color stability, Hybrid ceramic, Profilometer, Spectrophotometer

## Abstract

**Background:**

The aim of this study is to evaluate the color stability of hybrid ceramics stored in different combinations of beverages that are routinely consumed.

**Methods:**

The specimens were prepared with resin nano-ceramic (Lava Ultimate, 3M Espe, USA) and hybrid ceramic (Vita Enamic, Vita Zahnfabrik, Germany). The specimens were aged in a thermocycler machine for 10,000 cycles. They were stored in different combinations of beverages (water, tea, coke, coffee, red wine, pomegranate juice, and turnip juice) for 12 + 12 h. Surface roughness measurements were performed using a profilometer. The specimen colors were measured using a spectrophotometer. The color values (L-a-b) of the specimens and mean surface roughness values (Ra) were recorded at the end of the 1st (D1), 7th (D7), 14th (D14) and 28th (D28) d.

**Results:**

When the solution groups were examined, significant color changes were observed in the Lava coffee-tea, Enamic coffee-tea, Lava coffee-wine, and Enamic coffee-wine groups compared with the other groups (*p* < 0.01). Except for the samples in the Lava coffee-wine 28th day (D28) and Enamic coffee-wine 28th day (D28) groups, more color changes were observed in the Lava samples than in the Enamic samples across all groups and periods.

**Conclusion:**

Greater color changes (except for the samples stored in coffee-wine) were observed in the Lava samples than in the Enamic samples across all groups and periods. It was observed that the coffee-tea and coffee-wine beverage combinations produced the greatest color change in hybrid ceramics within the limitations of this study.

## Introduction

The successful sustenance of a well-prepared restoration is possible if its physical, mechanical, and esthetic properties are maintained. High strength hybrid ceramics prepared using computer-aided manufacturing are gaining increasing popularity [1], owing to their superior biocompatibility, improved mechanical properties, and low plaque accumulation on their surfaces [2, 3].

Moreover, hybrid ceramics are considered successful in mimicking dentin with ease of milling and adjusting [4]. Lava Ultimate was launched in 2011 [5] and Vita Enamic was launched in 2013 [6]. In this respect, hybrid ceramics provide ease of use for dental clinicians and technicians, in terms of both inlay-onlay and crown restorations.

The color stability of the restoration is the most sought-after esthetic property. The degree of discoloration depends mainly on the material composition, solution type and exposure time [7]. In studies regarding the ceramic color stability, mostly tea, cola, and coffee were used as coloring agents [8, 9]. Thermal aging is a frequently used and reliable method for simulating intraoral thermal change conditions that undergone by a restoration has for in vitro studies [10, 11]. Studies have shown that artificial aging by thermal cycling and exposure to colored beverages have a significant effect on the optical properties of resin-based ceramic materials, thereby affecting the aesthetic outcome [12, 13].

The classic CIELab color formula developed by the Commission of Internationale de l’Eclairage (CIE) includes lightness, hue, and chroma, represented by L*, a*, and b*, respectively. L* represents lightness and takes a value between 0 and 100 [14], where 0 represents black and 100 represents white. a* refers to the saturation on the red-green axis, and b* refers to the saturation on the yellow-blue axis. Color differences were calculated using these parameters as ∆E values using a formula. The CIE introduced the CIEDE2000 color formulation in 2000 with the addition of two new parameters [15]. The CIEDE 2000 color system is widely used in the color measurement of ceramics [16].

One of the most important issues in determinig the color stability is the surface roughness of the studied material. Among the factors that cause roughening of the surface of restorative materials in the oral environment are the nutrition type, salivary flow, temperature difference, and bacterial flora diversity of the environment. It has been proven that the method of measuring surface roughness with a profilometer gives successful results. The measurement of mean Ra value as 0.3 µm, is granted as the clinically acceptable limit in literature [17].

Coffee consumption is common in worldwide. Global coffee consumption in 2019/20, according to the International Coffee Organization (ICO), was 0.7% higher than in 2018/19 [18]. Beverage habits vary in societies in line with individual preferences. More than one drink is often included in daily diet. Thus, the difference between our study and other coloring studies is that many drinks were evaluated together, as in our daily lives. The first null hypothesis tested indicated that the storage with different combinations of water, coffee, tea, coke, red wine, pomegranate juice, and turnip juice would not cause a color change in both hybrid ceramics, and the second null hypothesis tested that exposure of hybrid ceramics to coloring beverages for different durations (1,7, 14, and 28 d) would not have any effect on discoloration. The third null hypothesis was that storage with different colorant beverage combinations would not cause any change in surface roughness in both hybrid ceramics. The fourth null hypothesis was that; exposure of hybrid ceramics to beverage combinations for different durations (1,7, 14, and 28 d) would not affect surface roughness.

## Materials and methods

**Fig. 1:**
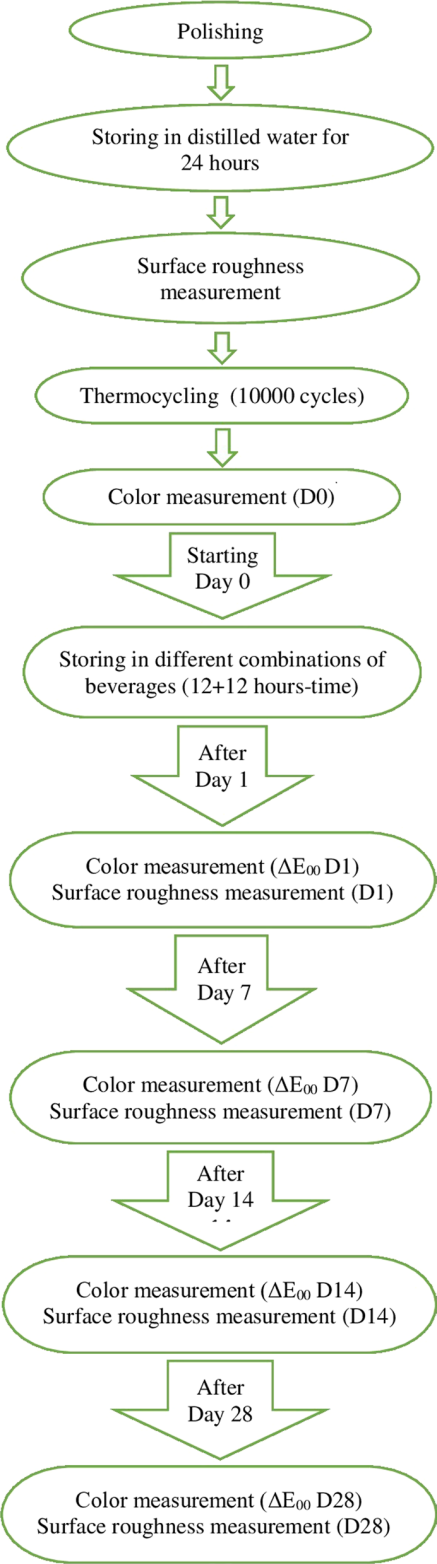
Diagram of the study design

The ceramics, polishing materials, and their properties used in the study are listed in Table 1. The study design is illustrated in Fig. 1. For preparing the samples in this study, both 14 mm. length hybrid ceramic blocks, A2-HT colored-Lava Ultimate (3M Espe, Minnesota, USA) and HT/2M2 colored Vita Enamic (Vita Zahnfabrik, Germany) were used.

**Table 1:** The materials and their properties used in the study

Material	Composition	Translucency/shade	Lot number	Manufacturer
Vita Enamic	Feldspathic ceramic (86%), acrylic polymer (14%)	HT/2M2	51040	Vita Zahnfabrik
Lava Ultimate	Resin nanoceramic (79%), polymer matrix (21%)	HT/A2	33140	3M ESPE
Shofu Super Snap Rainbow Technique Kit	Silicon carbide, aluminum carbide	–	0715012	Shofu Dental Corporation

Specimens of 1 mm were obtained using a precision cutting device (Metkon Industrial Equipment, Istanbul, Turkey). All specimens were then polished using a polishing set (Shofu Super Snap Rainbow Technique Kit, Shofu Dental Corporation, San Marcos, USA). The polishing procedure was performed in the following order: polishing with a super-snap disk at 15,000 rpm, polishing with dura white-stone at 30,000 rpm, and polishing with a composite fine-disk at 20,000 rpm, according to the manufacturer’s recommendations. All polishing procedures were performed on both sides of the samples using a low-speed handpiece (Kavo Ewl 4990; Kavo Dental GmbH, Germany) by the same investigator. After polishing, all specimens were stored in distilled water for 24 h.

The samples were aged in distilled water baths at 5 °C and 55 °C with a dwell time of 30 s for 10,000 cycles in a thermocycler machine (Thermocycler THE1100, SD Mechatronic GmbH, Feldkirchen-Westerham, Germany), that equivalent to using the restoration in the mouth for one year.

It is believed that most individuals drink coffee on a daily basis. Therefore, coffee was preferred in beverage combinations in all groups, except in the water-water group. Different groups were formed by combining water, coffee, or other colored drinks with coffee [19]. The specimens were randomly divided into eight groups (n = 8 each). They were stored in different beverage combinations for 12 + 12 h. The beverages used in this study are listed in Table 2. The groups tested with different beverage combinations are listed in Table 3. The samples were kept in closed containers to prevent evaporation of the staining solutions throughout the study. The beverages were renewed every 12 h. The specimens were placed in an incubator (Incubator EN 25 032 Core, Core Materials Manufacturing Industry & Trade Co., Istanbul, Turkey) at 37 °C, with the exception of solution changes and measurements. Before color measurements, the samples were washed under running water for 10 s and then dried using a tissue.

**Table 2:** The beverages used in the study

Beverage	Preparation	Manufacturer
Water	–	Distilled water
Tea	One tea bag was put in 150 ml of boiled water for 5 min	Lipton Co., Rize, Turkey
Coke	–	Coca Cola Co., Istanbul, Turkey
Coffee	10 g of coffee powder was put into 500 ml of boiled distilled water. After 10 min of stirring, coffee solution was filtered through a filter paper	Nescafe Classic, Nestle, Bursa, Turkey
Red wine	–	Mediterranean pearl, Deva Wine Co., Manisa, Turkey
Pomegranate juice	It is obtained from pomegranate fruit by squeezing method (250 ml of pomegranate juice was obtained from 1 kg of pomegranate)	–
Turnip juice	–	Dimes Food Co., Izmir, Turkey

**Table 3:** The beverage combinations used in our study and the names of the experimental groups

Group no	Beverages (12–12 h)	Group name	
		Lava Ultimate (Lava)	Vita Enamic (Enamic)
1	Water–water	Lava water–water	Enamic water–water
2	Coffee–water	Lava coffee–water	Enamic coffee–water
3	Coffee–coffee	Lava coffee–coffee	Enamic coffee–coffee
4	Coffee–tea	Lava coffee–tea	Enamic coffee–tea
5	Coffee–coke	Lava coffee–coke	Enamic coffee–coke
6	Coffee–wine	Lava coffee–wine	Enamic coffee–wine
7	Coffee–pomegranate juice	Lava coffee–pomegranate juice	Enamic coffee–pomegranate juice
8	Coffee–turnip juice	Lava coffee–turnip juice	Enamic coffee–turnip juice

L, a, and b values were determined using a spectrophotometer (Vita Easy Shade Advance 4.0, Vita Zahnfabrik, Bad Säckingen, Germany). One surface of each sample was marked with a diamond round bur. Color change measurements were performed on the unmarked side under standard D65 lighting conditions and on a white background at the end of the 1st (D1), 7th (D7), 14th (D14), and 28th (D28) d. In the literature, keeping the samples in beverages for one day in the in vitro environment is considered equivalent to the sample exposed to a beverage for one month in the clinical environment [20, 21]. The ΔE_00_ values were calculated using the CIEDE 2000 color-difference formula.

$$\Delta E_{00} = \left( {\left( {\frac{{\Delta L^{*} }}{{k_{{\text{L}}} S_{{\text{L}}} }}} \right)^{2} + \left( {\frac{{\Delta C^{\prime}}}{{k_{{\text{C}}} S_{{\text{C}}} }}} \right)^{2} + \left( {\frac{{\Delta H^{\prime}}}{{k_{{\text{H}}} S_{{\text{H}}} }}} \right)^{2} + R_{T } \left( {\frac{{\Delta C^{\prime}\Delta H^{\prime}}}{{S_{{\text{C}}} S_{{\text{H}}} }}} \right)} \right)1/2$$

In addition, the surface roughness of the samples was measured using a profilometer (Taylor Hobson Surtronic 25, Leicester, UK) with a 0.25 mm cut-off value at the end of the 1st (D1), 7th (D7), 14th (D14), and 28th (D28) d. As mentioned previously, one surface of each sample was marked. The surface roughness measurements were performed on the unmarked side. The average roughness values were calculated for three different points on each sample surface. A constant measuring speed of 0.5 mm/sec was used to determine the average surface roughness profile (Ra) in µm.

### Statistical analysis

The sample size calculation was performed using G*Power v. 3.1.9.3 software (Heinrich-Heine-Universität Düsseldorf, Germany). A sample size, at the level of *α* = 0.05, with an effect size of 0.6 and power of 0.8 was used. Accordingly, a total of at least 48 samples, six in each group (n = 6), should be studied.

Statistical analyses were performed using IBM SPSS Statistics for Windows (version 22.0, IBM Corp., Armonk, NY, USA). Descriptive statistics have been presented as the mean ± standard deviation (SD) for continuous data. The normality of distribution was assessed using the Shapiro–Wilk test. The Mann Whitney U test was used for non‐normally distributed data in continuous variable comparisons between the two groups. The Kruskal–Wallis test was used for non‐normally distributed data in continuous variable comparisons among more than two groups. After the Kruskal–Wallis test, Mann–Whitney U tests with the Bonferroni correction post hoc multiple comparison test were used to determine the group that caused the difference. Pearson’s correlation test was performed to analyze the relationship between the amount of color change and roughness change. A *p* value < 0.05 was considered statistically significant.

## Results

**Fig. 2:**
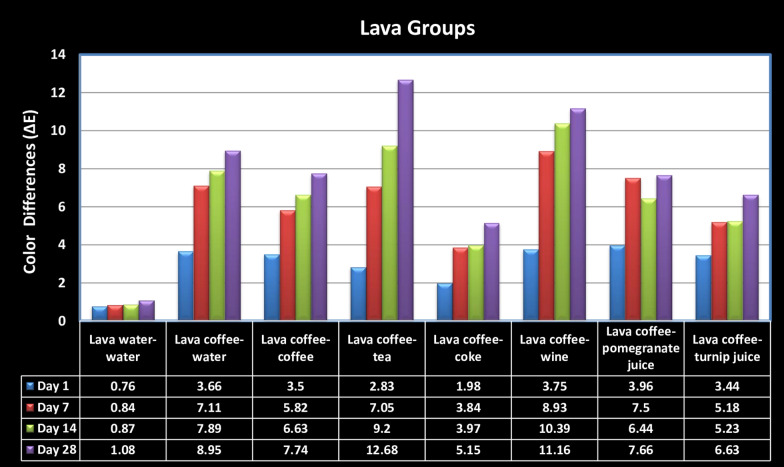
Graph of the color differences (ΔE_00_) on Lava groups due to time

**Fig. 3:**
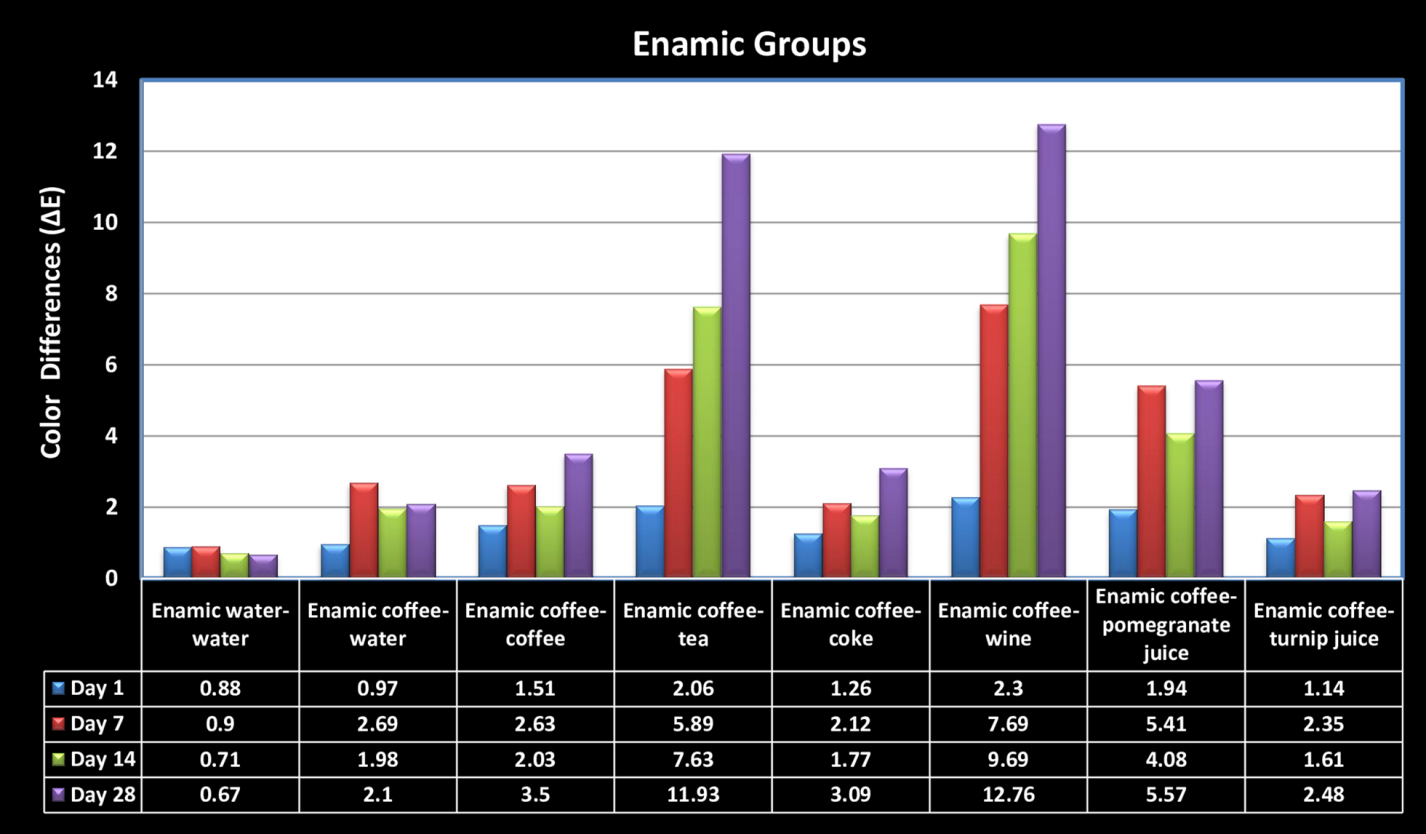
Graph of the color differences (ΔE_00_) on Enamic groups due to time

Descriptive statistics of color difference (ΔE_00_) values for each hybrid ceramic after 1st (D1), 7th (D7), 14th (D14), and 28th (D28) day of storage for each beverage combination are listed in Table 4. Furthermore, the graphs of color differences in the Lava groups (Fig. 2) and the Enamic groups based on time are presented (Fig. 3). The surface roughness (Ra) values for each hybrid ceramic after 1st (D1), 7th (D7), 14th (D14), and 28th (D28) day of storage in each beverage combination are listed in Table 5.

**Table 4:** Descriptive statistics of color differences (ΔE_00_) values for each groups after 1 day (D1), 7 day (D7), 14 day (D14), 28 day (D28) of stored in each beverage combination

Time		Lava groups	Mean ± SD	Enamic groups	Mean ± SD	*p1*
D1		Lava water–water	0.76 ± 0.49^*(A)*^	Enamic water–water	0.88 ± 0.33^*(A)*^	0.443
D7			0.84 ± 0.19^*(A)*^		0.9 ± 0.18^*(A)*^	0.701
D14			0.87 ± 0.32^*(A)*^		0.71 ± 0.44^*(A)*^	0.277
D28			1.08 ± 0.4^*(A)*^		0.67 ± 0.41^*(A)*^	0.048*
*p*2			0.239		0.319	
D1		Lava coffee–water	3.66 ± 1.74^*(a,B)*^	Enamic coffee–water	0.97 ± 0.12^*(a,A)*^	0.011*
D7			7.11 ± 3.21^*(ab,C)*^		2.69 ± 0.59^*(b,B)*^	0.035*
D14			7,89 ± 3.71^*(bc,BD)*^		1.98 ± 0.32^*(b,BD)*^	0.002*
D28			8.95 ± 4.24^*(c,B)*^		2.1 ± 0.24^*(b,AB)*^	0.002*
*p2*			< 0.001**		0.002**	
D1		Lava coffee–coffee	3.5 ± 1.06^*(a,B)*^	Enamic coffee–coffee	1.51 ± 0.19^*(a,BC)*^	0.002*
D7			5.82 ± 1.75^*(ab,BC)*^		2.63 ± 0.28^*(ab,B)*^	0.009*
D14			6.63 ± 2.26^*(bc,BD)*^		2.03 ± 0.23^*(bc,BD)*^	0.002*
D28			7.74 ± 2.43^*(c,B)*^		3.5 ± 0.42^*(c,CE)*^	0.004*
*p2*			< 0.001**		< 0.001**	
D1		Lava coffee–tea	2.83 ± 0.97^*(a,AB)*^	Enamic coffee–tea	2.06 ± 0.49^*(a,CD)*^	0.179
D7			7.05 ± 1.58^*(ab,C)*^		5.89 ± 0.57^*(ab,C)*^	0.180
D14			9.2 ± 1.83^*(bc,BD)*^		7.63 ± 0.77^*(bc,C)*^	0.085
D28			12.68 ± 1.33^*(c,C)*^		11.93 ± 1.01^*(c,D)*^	0.406
*p2*			< 0.001**		< 0.001**	
D1		Lava coffee–coke	1.98 ± 1^*(a,AB)*^	Enamic coffee–coke	1.26 ± 0.23^*(a,AB)*^	0.250
D7			3.84 ± 1.55^*(ab,AB)*^		2.12 ± 0.38^*(ab,AB)*^	0.035*
D14			3.97 ± 1.63^*(bc,AD)*^		1.77 ± 0.44^*(bc,AB)*^	0.004*
D28			5.15 ± 2.14^*(c,AB)*^		3.09 ± 0.38^*(c,BC)*^	0.110
*p2*			< 0.001**		< 0.001**	
D1		Lava coffee–wine	3.75 ± 1.09^*(a,B)*^	Enamic coffee–wine	2.3 ± 0.38^*(a,D)*^	0.006
D7			8.93 ± 2.09^*(ab,C)*^		7.69 ± 1.44^*(ab,C)*^	0.225
D14			10.39 ± 2.3^*(bc,CB)*^		9.69 ± 2.09^*(bc,C)*^	0.482
D28			11.16 ± 3.2^*(c,C)*^		12.76 ± 1.72^*(c,D)*^	0.250
*p2*			< 0.001**		< 0.001**	
D1		Lava coffee–pomegranate juice	3.96 ± 1.16^*(a,B)*^	Enamic coffee–pomegranate juice	1.94 ± 0.3^*(a,CD)*^	0.002*
D7			7.5 ± 1.28^*(b,C)*^		5.41 ± 1.39^*(bc,C)*^	0.013*
D14			6.44 ± 1.44^*(c,BD)*^		4.08 ± 0.94^*(ab,CD)*^	0.013*
D28			7.66 ± 1.61^*(b,B)*^		5.57 ± 0.73^*(c,CD)*^	0.006*
*p2*			< 0.001**		< 0.001**	
D1		Lava coffee–turnip juice	3.44 ± 0.64^*(a,B)*^	Enamic coffee–turnip juice	1.14 ± 0.47^*(a,AB)*^	0.002*
D7			5.18 ± 1.3^*(b,BC)*^		2.35 ± 0.45^*(bc,AB)*^	0.003*
D14			5.23 ± 1.6^*(b,D)*^		1.61 ± 0.57^*(ab,AB)*^	0.002*
D28			6.63 ± 1.89^*(c,B)*^		2.48 ± 0.25^*(c,ABE)*^	0.002*
*p2*			< 0.001**		< 0.001**	
*p3*	D1	0.001***		< 0.001***		
	D7	< 0.001***		< 0.001***		
	D14	< 0.001***		< 0.001***		
	D28	< 0.001***		< 0.001***		

*p1*: The significance of the color change differences between hybrid ceramic groups at the same beverage groups and same duration times (*: *p*1 < 0.05)

*p2*: The significance of the color change differences between different duration times in the same beverage group for each ceramic group (**: *p*2 < 0.05). Lowercase letters used for multiple comparisons (a,b,c)

*p3*:The significance of the color change differences between different beverage groups at the same time intervals for each ceramic group (***: *p*3 < 0.05). Capital letters used for multiple comparisons (A,B,C,D,E)

The mean difference (*p*) was set significant at the 0.05 level

Similar superscript letters indicate statistically indifference between groups (*p* < 0.05)

Dissimilar superscript letters indicate statistically significant differences between groups (*p* < 0.05)

The two-way analysis of variance in repeated measures

*p1*: Mann Whitney U test

*p2*: Friedman F test

*p3*: Kruskal Wallis variance test

**Table 5:** Descriptive statistics of surface roughness (Ra) values (µm) for each groups after 1 day (D1), 7 day (D7), 14 day (D14), 28 day (D28)

Time		Lava groups	Mean ± SD	Enamic groups	Mean ± SD	*p1*
D1		Lava water–water	0.16 ± 0.03	Enamic water–water	0.16 ± 0.02	0.740
D7			0.19 ± 0.04		0.15 ± 0.03^*(A)*^	0.042*
D14			0.21 ± 0.04		0.15 ± 0.01^*(A)*^	0.006*
D28			0.16 ± 0.06		0.14 ± 0.01^*(A)*^	0.999
*p2*			0.058		0.198	
D1		Lava coffee–water	0.15 ± 0.03^*(a)*^	Enamic coffee–water	0.17 ± 0.03^*(ac)*^	0.296
D7			0.16 ± 0.07^*(a)*^		0.24 ± 0.07^*(b,BC)*^	0.109
D14			0.3 ± 0.15^*(b)*^		0.29 ± 0.09^*(b,BC)*^	0.520
D28			0.16 ± 0.04^*(a)*^		0.15 ± 0.03^*(c,AC)*^	0.895
*p2*			0.020**		0.001**	
D1		Lava coffee–coffee	0.15 ± 0.06^*(a)*^	Enamic coffee–coffee	0.17 ± 0.03	0.189
D7			0.16 ± 0.04^*(a)*^		0.18 ± 0.03^*(AC)*^	0.374
D14			0.28 ± 0.11^*(b)*^		0.21 ± 0.05^*(AC)*^	0.246
D28			0.18 ± 0.07^*(ab)*^		0.2 ± 0.05^*(BC)*^	0.520
*p2*			0.022**		0.077	
D1		Lava coffee–tea	0.16 ± 0.05^*(a)*^	Enamic coffee–tea	0.16 ± 0.02^*(a)*^	0.896
D7			0.17 ± 0.04^*(ab)*^		0.25 ± 0.1^*(b,BC)*^	0.039*
D14			0.35 ± 0.18^*(b)*^		0.33 ± 0.08^*(b,B)*^	0.847
D28			0.15 ± 0.05^*(a)*^		0.21 ± 0.07^*(ab,BD)*^	0.080
*p2*			0.031**		0.013**	
D1		Lava coffee–coke	0.15 ± 0.04	Enamic coffee–coke	0.18 ± 0.07	0.474
D7			0.19 ± 0.06 ara>		0.17 ± 0.03^*(AC)*^	0.561
D14			0.31 ± 0.22		0.26 ± 0.1^*(BC)*^	0.949
D28			0.17 ± 0.03		0.21 ± 0.04^*(BD)*^	0.106
*p2*			0.133		0.088	
D1		Lava coffee–wine	0.15 ± 0.04	Enamic coffee–wine	0.16 ± 0.02	0.358
D7			0.16 ± 0.05		0.17 ± 0.06^*(AD)*^	0.948
D14			0.25 ± 0.11		0.32 ± 0.24^*(BC)*^	0.898
D28			0.15 ± 0.02		0.18 ± 0.05^*(ACD)*^	0.265
*p2*			0.056		0.066	
D1		Lava coffee–pomegranate juice	0.15 ± 0.04	Enamic coffee–pomegranate juice	0.16 ± 0.03^*(a)*^	0,422
D7			0.19 ± 0.05		0.21 ± 0.05^*(a,BCD)*^	0,897
D14			0.24 ± 0.09		0.36 ± 0.13^*(b,B)*^	0,034*
D28			0.16 ± 0.05		0.21 ± 0.03^*(a,BD)*^	0,077
*p2*			0.177		0.001**	
D1		Lava coffee–turnip juice	0.15 ± 0.04^*(a)*^	Enamic coffee–turnip juice	0.17 ± 0.02^*(a)*^	0.304
D7			0.22 ± 0.12^*(a)*^		0.2 ± 0.04^*(a,BCD)*^	0.949
D14			0.37 ± 0.18^*(b)*^		0.36 ± 0.17^*(b,B)*^	0.948
D28			0.17 ± 0.04^*(a)*^		0.2 ± 0.04^*(a,BC)*^	0.158
*p2*			0.002**		0.021**	
*p3*	D1	0.988		0.924		
	D7	0.686		0.009***		
	D14	0.332		0.003***		
	D28	0.922		0.016***		

*p1*:The significance of surface roughness differences between hybrid ceramic groups at the same beverage groups and same duration times (*: *p*1 < 0.05)

*p2*:The significance of the surface roughness differences between different duration times in the same beverage group for each ceramic group (**: *p*2 < 0.05). Lowercase letters used for multiple comparisons (a,b,c)

*p3*:The significance of surface roughness differences between different beverage groups at the same time intervals for each ceramic group (***: *p*3 < 0.05). Capital letters used for multiple comparisons (A,B,C,D)

The mean difference (*p*) was set significant at the 0.05 level

Similar superscript letters indicate statistically indifference between groups (*p* < 0.05)

Dissimilar superscript letters indicate statistically significant differences between groups (*p* < 0.05)

The two-way analysis of variance in repeated measures

*p1*: Mann Whitney U test

*p2*: Friedman F test

*p3*: Kruskal Wallis variance test

The highest color difference (12.68 ± 1.33) in the Lava groups was in the Lava coffee-tea group, which was stored for 28 d. The highest color difference (12.76 ± 1.72) in the Enamic groups was in the Enamic coffee-wine group, which was stored for 28 d. When the solution groups were examined, significantly more color changes were observed in the Lava coffee-tea, Enamic coffee-tea, Lava coffee-wine, and Enamic coffee-wine groups than in other groups (*p* < 0.01). When the color changes between the Lava and Enamic samples were compared, except for the samples in the Lava coffee-wine and Enamic coffee-wine groups, which were stored for 28 d, more color changes were observed in the Lava samples than in the Enamic samples across all groups and periods. Statistically significant color changes were observed in the Lava coffee-water, Lava coffee-coffee, Lava coffee-pomegranate juice, and Lava coffee-turnip juice groups compared with the Enamic coffee-water, Enamic coffee-coffee, Enamic coffee-pomegranate juice, and Enamic coffee-turnip juice groups in each period (*p* < 0.05). In both materials, no significant color differences were observed in the Lava water-water and Enamic water-water groups over time. Except for the Enamic coffee-water group, the least color change was observed on the 1st day and the greatest color change was observed on the 28th day in all solution groups (*p* < 0.001).

The highest roughness value (Ra) among the Lava samples was observed on the 14th day in the Lava coffee-turnip juice group (0.37 ± 0.18 µm). The highest roughness values (Ra) among the Enamic samples were observed in the Enamic coffee-pomegranate juice and Enamic coffee-turnip juice groups as 0.36 ± 0.13 µm and 0.36 ± 0.17 µm, respectively, on the 14th day (*p* < 0.05). When each group was evaluated within itself, the highest in-group roughness values were measured on the 14th day in all groups, except the Enamic water-water group. This difference was statistically significant in the Lava coffee-water, Lava coffee-coffee, Lava coffee-tea, Lava coffee-turnip juice, Enamic coffee-water, Enamic coffee-tea, Enamic coffee-pomegranate juice, Enamic coffee-turnip juice groups (*p* < 0.05). When analyzed according to the same periods; there was no statistically significant difference in roughness values between the Lava groups in any of the different time groups (*p* > 0.05). Furthermore, statistically significant difference was observed in the roughness values between the groups in the measurements made on the 1st day in the Enamic group (*p* > 0.05). However, the roughness values were significantly different among different Enamic groups in the measurements made on the 7th,14th, and 28th days (*p* < 0.05).

The correlation test results between the amount of color change and roughness change values are presented in Table 6. A positive correlation is observed between color change and roughness values on the Lava coffee-wine 7th day, Lava coffee-turnip juice 1st and 7th day groups (positive correlation: when one of the variable value increases, the other variable value also increases and vice versa). A negative correlation, however, was observed between the color change and roughness values in the Lava coffee-coke 7th day, Enamic coffee-tea 1st day groups (negative correlation: when values increase in one variable, the value of the other variable decreases).

**Table 6:** Correlations between color change and surface roughness values on different days between groups

Time	Lava groups	r	*p*	Enamic groups	r	*p*
D1	Lava water–water	0.297	0.517	Enamic water–water	0.006	0.990
D7		0.049	0.917		− 0.535	0.216
D14		0.638	0.123		− 0.287	0.533
D28		0.752	0.051		− 0.507	0.245
D1	Lava coffee–water	− 0.287	0.532	Enamic coffee–water	− 0.020	0.967
D7		0.403	0.370		− 0.029	0.950
D14		0.572	0.179		− 0.352	0.438
D28		0.497	0.256		0.185	0.691
D1	Lava coffee–coffee	− 0.023	0.961	Enamic coffee–coffee	0.072	0.877
D7		0.294	0.523		0.353	0.438
D14		0.019	0.968		− 0.350	0.442
D28		− 0.329	0.471		0.194	0.677
D1	Lava coffee–tea	− 0.212	0.648	Enamic coffee–tea	− 0.829*	0.021*
D7		− 0.576	0.176		− 0.479	0.276
D14		0.169	0.718		− 0.487	0.267
D28		0.071	0.880		− 0.269	0.560
D1	Lava coffee–coke	− 0.322	0.481	Enamic coffee–coke	− 0.090	0.848
D7		− 0.898**	0.006**		0.002	0.996
D14		0.082	0.861		− 0.239	0.606
D28		− 0.450	0.311		0.024	0.960
D1	Lava coffee–wine	− 0.254	0.582	Enamic coffee–wine	− 0.196	0.673
D7		0.760*	0.048*		0.016	0.973
D14		− 0.737	0.059		− 0.408	0.364
D28		− 0.609	0.147		− 0.449	0.312
D1	Lava coffee–pomegranate juice	0.674	0.097	Enamic coffee–pomegranate juice	− 0.421	0.347
D7		− 0.475	0.282		0.628	0.131
D14		0.008	0.986		− 0.056	0.905
D28		− 0.392	0.384		0.230	0.619
D1	Lava coffee–turnip juice	0.800*	0.031*	Enamic coffee–turnip juice	− 0.410	0.361
D7		0.803*	0.030*		0.618	0.139
D14		− 0.334	0.464		0.519	0.233
D28		0.217	0.640		0.109	0.815

r: Pearson’s correlation coefficient

*Interpretation of the correlation coefficient (r):*

r < 0.2; very weak correlation or no correlation

r = 0.2–0.4; weak correlation

r = 0.4–0.6; moderate correlation

r = 0.6–0.8; high correlation

r > 0.8; very high correlation

**Correlation is significant at the *p* < 0.01 level

*Correlation is significant at the *p* < 0.05 level

## Discussion

Some beverages reportedly cause more staining in composite and ceramic restorations. It has been proven in many color stability studies on resin and hybrid ceramic materials that coffee is the most coloring beverage [11, 22, 23]. In our study, in which coffee was accepted as the main coloring beverage, the highest staining was observed after 28 d for Lava after storage in coffee-tea (∆E = 12.68) and for Enamic after storage in coffee-wine (∆E = 12.68) among all groups.

The first null hypothesis in this study was partially rejected because storage with different combinations of water, coffee, tea, cola, red wine, pomegranate juice, and turnip juice had a significant effect on color change in both hybrid ceramics.

The hybrid resin matrix falls into the dental ceramics category because it contains more than 50% inorganic particles [24]. Ceramics are inert, but their organic structure is weak. Coffee can easily diffuse into the organic matrix of the hybrid material depending on the water absorption capacity. In addition, the water absorption capacity of BIS-GMA has been reported to be higher than that of UDMA, TEGDMA, and BIS-EMA [25, 26]. Lava contains BIS-GMA, as distinct from Enamic. In the present study, a comparison of the color changes between Lava and Enamic samples (except for the samples in the Lava coffee-wine and Enamic coffee-wine groups) demonstrated more color changes in the Lava samples than in the Enamic samples across all groups and periods.

To better reflect the intraoral thermal exchange conditions, the samples were placed in a thermal cycler device (10,000 cycles) in our study. Lava and Enamic samples were placed in coffee for 5000 thermal cycles in a study by Acar et al. [11]. In this study, the highest color change was observed in the nanocomposite resin (Filtek Supreme Ultra Universal), followed by nanoceramic resin (Lava Ultimate), hybrid ceramic (Vita Enamic), and lithium disilicate ceramic (IPS e.max CAD), similar to our study. The study differed from our study in that the specimens were polished with silicon carbide abrasive papers only, and coffee was used for staining every 8 h and replaced with fresh coffee. In addition, the specimens were brushed with toothpaste after thermal aging.

Sagsoz et al. [27] investigated the staining resistance of CAD/CAM block (including Lava Ultimate and Vita Enamic) surfaces polished with different polishing kits. The specimens were immersed in tea, Turkish coffee, fermented black carrot juice, and distilled water for one day, one week, and one month. A month later, unpolished Lava specimens were found to have dramatically higher ∆E values than unpolished Enamic specimens. This result could be attributed to water absorption by the monomers. No thermal aging protocol was used in this study. In addition, based on the study by Ertaş et al. [20], it was reported that the one month immersion period was equivalent to 2.5 years of clinical aging in the study.

In the present study, no significant color differences were observed in the Lava water-water and Enamic water-water groups depending on the time in both hybrid ceramics. However, the least color change was observed on the 1st day, and the greatest color change was observed on the 28th day in all solution groups, except the Enamic coffee-water group (*p* < 0.001). The second null hypothesis of the research was partially accepted because it was found that the storage of hybrid ceramics in beverage combinations for different periods had a significant effect on the color change.

We consume various types of drinks in succession throughout the day routinely, which can cause discoloration of the teeth or restorations. However, a single type of colored beverage does not reflect the staining potential of human feeding behavior. In the literature, there are studies based on the pH of coloring beverages [19]. It is perceived that low-pH beverages will increase staining owing to the surface abrasion [28].

Arocha et al. [29] investigated the color stainability of Lava Ultimate, Paradigm, and two indirect laboratory-processed composites after immersion in coffee, red wine, black tea, and distilled water for four weeks. Staining solutions were renewed every two days, but without any thermal aging protocol. Similar to the results of our study, the ∆E_00_ values of the Lava Ultimate specimens immersed in red wine were higher than those immersed in coffee, black tea, and distilled water. Based on previous studies, it was concluded that this result may facilitate staining by softening the resin matrix with alcohol. The ∆E_00_ values (11.16) of the Lava specimens in the Lava coffee-wine group (statistically not different from the specimens in Lava coffee-tea) after the 28th day were higher than those of the others in our study.

In our study, color measurements were made using the Easyshade device, which has been reported as the most accurate color measurement device (92.6%) in the literature [30, 31]. The CIEDE2000 color formula has been determined to be better for color differences that are perceivable with the human eye than the CIELab color formula [32]. A ∆E color difference between 1.5 and 3 was determined to be observable alteration (noticeable) on the National Bureau of Standards (NBS) system of expressing color differences [14]. Color differences of more than 3.3 are easily detectable by laymen but color differences less than 3.3, are detectable only by experts, as reported in a previous study [33].

It has been reported in the literature that the surface roughness affects the color stability of ceramics. Ra < 0.2 µm is preferred to prevent biofilm retention on restorations [34]. A 0.3 µm Ra is a generally accepted standard for creating a smooth restoration surface [34]. Moreover, for an existing Ra > 0.3 µm, patients reportedly experience discomfort and feel the restoration surface by their tongue or lip contact [17]. Hybrid ceramics have been reported to be more resistant than conventional ceramics in terms of surface roughness owing to extrinsic coloring agents [35]. In our study, the highest roughness value in the Lava samples was observed in the measurements made on the 14th day in the Lava coffee-turnip juice group (0.37 ± 0.18 µm). The highest roughness values in the Enamic samples were observed in the Enamic coffee–pomegranate juice and Enamic coffee–turnip juice groups as 0.36 ± 0.13 µm and 0.36 ± 0.17 µm, respectively, in the measurements made on the 14th day. The third and fourth null hypotheses were rejected, which is in line with these results. In addition, all recorded Ra values were less than 0.4 µm in the present study.

The biggest limitation of our study is that it was an in vitro study. Although temperature changes were simulated, a complete simulation was not possible because of several factors such as bacteria in the oral flora, the structure of saliva, and hygiene habits. Therefore, further in vivo studies are required.

However, the present study evaluated the color stability under in vitro conditions with storage combinations of different beverages, both of hybrid ceramics, A2-HT colored-Lava Ultimate, and HT/2M2 colored-Vita Enamic blocks. The fact that blocks of different shades were not used is also a limitation of this study. This study may provide insights into the surface roughness and color stability of these materials. Further in vivo studies are needed to determine long-term shade maintenance in the decision-making process of dental material selection.

## Conclusion

Based on the results of the present study, except for the samples stored in coffee-wine, more color changes were observed in the Lava samples than in the Enamic samples across all groups and periods. In addition, the highest surface roughness values were detected after 14 d for all samples. The combinations of coffee-tea and coffee-wine beverages are likely the most accepted coloring beverages for hybrid ceramics, within the limitation of this study.

## Data Availability

The datasets generated and/or analyzed during the current study are not publicly available due to ensure that the data of the study is more secure in the review process in this way, but are available from the corresponding author on reasonable request.

## Abbreviations

°: Degree
°C: Centigrade degrees
%: Percent
µm: Micrometer
Bis-EMA: Bisphenol A diglycidyl methacrylate ethoxylated
Bis-GMA: Bisphenol A diglycidyl methacrylate
CAD: Computer-aided design
CAM: Computer-aided manufacturing
d: Day
h: Hour
HT: High translucent
mm: Milimeter
Ra: Surface roughness
rpm: Revolutions per minute
s: Second
TEGDMA: Triethylene glycol dimethacrylate
UDMA: Urethane dimethacrylate
